# Controversies in the Treatment of Classical Hodgkin Lymphoma

**DOI:** 10.1097/HS9.0000000000000149

**Published:** 2018-09-27

**Authors:** Dennis A. Eichenauer, Marc André, Peter Johnson, Alexander Fossa, Olivier Casasnovas, Andreas Engert

**Affiliations:** 1 First Department of Internal Medicine, University Hospital Cologne, Cologne, Germany; 2 German Hodgkin Study Group (GHSG), University Hospital Cologne, Cologne, Germany; 3 Department of Hematology, Université Catholique de Louvain, CHU UCL Namur, Yvoir, Belgium; 4 Cancer Research UK Centre, University of Southampton, Southampton, UK; 5 Department of Oncology, Oslo University Hospital, Oslo, Norway; 6 Department of Hematology, CHU Dijon, Dijon, France.

## Abstract

Hodgkin lymphoma (HL) is a B-cell-derived malignancy that mostly affects young adults. Pathologically, HL is divided into classical HL (cHL) and the rare entity of nodular lymphocyte-predominant HL. Classical HL is characterized by few malignant cells termed Hodgkin and Reed–Sternberg cells embedded in an inflammatory background. The treatment of cHL has consistently improved over the last decades so that current standard approaches result in long-term remission rates in excess of 80%. However, potentially lethal therapy-related late complications affect an increasing number of survivors. For this reason, issues regarding the optimal treatment of cHL patients are still fiercely debated. Questions under discussion include how treatment can be guided by interim positron emission tomography, the best initial treatment for advanced-stage disease and the use of targeted drugs such as the antibody–drug conjugate brentuximab vedotin and the anti-PD-1 antibodies nivolumab and pembrolizumab. The identification of patients who should undergo allogeneic stem cell transplantation is another unsolved issue. The present article highlights the most relevant clinical trials and addresses controversial open questions in the treatment of cHL.

## Introduction

Hodgkin lymphoma (HL) is a B-cell-derived lymphoid malignancy with an incidence of 2 to 3/100.000/year in the Western world. Approximately 95% of all HL patients are diagnosed with classical HL (cHL) while 5% of cases present with the distinct entity of nodular lymphocyte-predominant HL. Steady improvement in the first-line treatment of cHL has been based on risk-adapted multiagent chemotherapy followed by radiotherapy (RT) in most patients. The definition of risk groups is based on the stage according to the Ann-Arbor classification and additional clinical parameters, and varies to some extent between research groups (Table 1). Risk-adapted therapy results in long-term remission rates that are currently exceeding 80% irrespective of the stage at diagnosis.^1^ Despite the success in curing cHL, however, chemotherapy and RT cause severe and potentially lethal late complications such as cardiovascular disease and second malignancies in a substantial minority of patients.^2–4^ Thus, the balance between cure and toxicity has been a main issue in the development of improved treatment strategies for cHL patients. In recent years, response-adapted therapy based on interim positron emission tomography (PET) has been studied to reduce toxicity whenever possible. However, there are several unsolved controversies in connection with interim PET, including its optimal use in the treatment of patients with early and intermediate stages. It is unclear whether consolidation RT can be omitted in a defined patient population with early metabolic remission.^5,6^ The most appropriate initial chemotherapy, i.e., escalated BEACOPP (bleomycin, etoposide, doxorubicin, cyclophosphamide, vincristine, procarbazine, prednisone) or ABVD (doxorubicin, bleomycin, vinblastine, dacarbazine) for patients diagnosed with advanced-stage disease is another subject of discussion in the treatment of cHL.^7–10^ In patients with disease recurrence after first-line treatment, the accepted standard of care consists of high-dose chemotherapy followed by autologous stem cell transplantation (ASCT).^11^ However, this standard is based on 2 randomized trials with a total of less than 200 patients, and the optimal salvage regimen is still not defined.^11–15^ In addition, the role of PET before high-dose chemotherapy and the role of consolidation therapy after high-dose chemotherapy and ASCT has to be clarified.^16,17^ The antibody–drug conjugate brentuximab vedotin (BV) and the anti-PD-1 antibodies nivolumab and pembrolizumab have been approved for the treatment of patients either relapsing after high-dose chemotherapy and ASCT or unable to undergo such a procedure, but the most appropriate sequence for the administration of these drugs has not been evaluated to date.^18–20^ Lastly, the role of allogeneic stem cell transplantation (allo-SCT) in the era of targeted therapies has to be reappraised.^21,22^ To shed more light on the controversies in the treatment of cHL, the current article presents the standard approaches and addresses unsolved issues in the management of this disease.

**Table 1:**
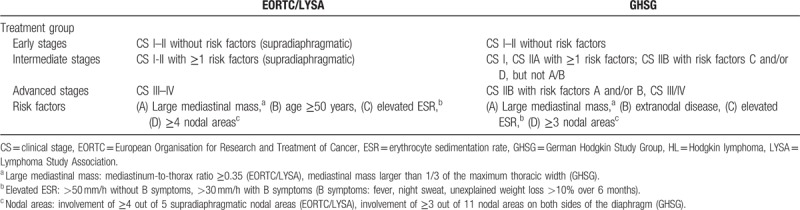
Definition of HL Risk Groups According to the EORTC/LYSA and the GHSG

## First-line treatment

### Early stages

The previous standard of care for patients with early-stage cHL (Table 1) consisted of a brief chemotherapy with 2 or 3 cycles of ABVD followed by limited-field RT. This standard was based on 2 prospective randomized trials: the German Hodgkin Study Group (GHSG) HD10 trial (comparing 2 cycles of ABVD followed by RT at 20 Gy, 2 cycles of ABVD followed by RT at 30 Gy, 4 cycles of ABVD followed by RT at 20 Gy and 4 cycles of ABVD followed by RT at 30 Gy) and the H8F study conducted by the European Organisation for Research and Treatment of Cancer (EORTC) and the Groupe d’Etudes des Lymphomes d’Adulte (GELA) (comparing 3 cycles of chemotherapy followed by RT and RT alone). According to the results of these large trials with a total of more than 1950 patients randomized, 95% of patients are still alive after 10 years of follow-up.^23,24^

Given the excellent outcome of cHL patients with early-stage disease, further improvements in progression-free survival (PFS) and overall survival (OS) no longer represent the major research goal. In contrast, possible ways to reduce treatment-related toxicity without compromising efficacy were investigated.

The H10 study was conducted by the EORTC, the Lymphoma Study Association (LYSA) and the Fondazione Italiana Linfomi (FIL) to evaluate omitting consolidation RT on the basis of an interim PET performed after 2 cycles of ABVD. A similar trial, using slightly different criteria for early-stage disease, was the RAPID study from the UK. After median observation times of 5.0 years in both studies, a slightly reduced tumor control was demonstrated for PET-negative patients (accounting for 87.0% of patients in the H10 study and 74.6% in the RAPID study when Deauville scores 1 and 2 were considered negative) who did not receive consolidation RT after chemotherapy (5-year PFS rates within the H10 study: 99.0% with consolidation RT vs 87.1% with chemotherapy alone; 3-year PFS rates within the RAPID study: 94.6% with consolidation RT vs 90.6% with chemotherapy alone), but no differences in OS were detected.^5,6^ The use of limited-field RT applied according to modern treatment standards may be less likely to cause severe and potentially lethal late effects, and the balance of risk between a small increase in recurrence and a small probability of late effects from RT will vary between patients, according to the localization of the lymphoma and patient risk factors for cardiovascular disease and second malignancies. However, extended follow-up of the H10 and RAPID studies is necessary to determine the treatment approach with the best long-term risk-benefit ratio.

Major controversy:

2 or 3 cycles of ABVD + limited-field RT (23, 24) vs ABVD only in patients with a negative interim PET (5, 6)

### Intermediate stages

Combined-modality treatment with 4 cycles of ABVD followed by limited-field RT has been the standard of care for cHL patients diagnosed with intermediate-stage disease (Table 1). According to the results of the GHSG HD14 study, the freedom from treatment failure (FFTF) at 5 years was 87.7% with an OS rate of 97.7%.^25^ Higher rates of failure-free disease control were achieved by further intensifying chemotherapy. In the experimental arm of the HD14 trial, 2 cycles of escalated BEACOPP were followed by 2 cycles of ABVD (“2 + 2”) before involved-field RT. This approach resulted in a better tumor control but was also more toxic when compared to the standard arm of the study using 4 cycles of ABVD (5-year FFTF rates: 94.8% with “2 + 2” followed by RT vs 87.7% with 4 cycles of ABVD followed by RT).^25^

More recent trials aimed at reducing toxicity ideally without compromising efficacy. Within the EORTC/LYSA/FIL H10 study, patients with intermediate-stage cHL who had a negative interim PET after 2 cycles of ABVD (accounting for 77.6% of study participants when Deauville scores 1 and 2 were considered negative) were randomized between 2 additional cycles of ABVD followed by limited-field RT or 4 additional cycles of ABVD without consolidation RT. After a median follow-up of 5.1 years, the final analysis of the study revealed a modest increase of the relapse rate among patients receiving chemotherapy alone (5-year PFS rates: 92.1% with consolidation RT vs 89.6% with chemotherapy alone) but no reduction in OS. Long-term data of the study are necessary to determine whether replacing state of the art RT by additional chemotherapy reduces the risk of long-term toxicity, and the balance of risk will vary between patients. The importance of extended observation has been underscored by the 12-year results of the randomized HD.6 study. After a median follow-up of 11.2 years, patients with limited-stage HL presenting with an unfavorable risk profile had a better OS after chemotherapy alone (4 or 6 cycles of ABVD) than after chemotherapy (2 cycles of ABVD) followed by subtotal nodal RT (92% vs 81%) although the freedom from disease progression rate was superior after combined-modality treatment (94% vs 86%).^26^ However, it has to be kept in mind that the large RT fields used in the HD.6 study that was conducted in the pre-PET era no longer represent the standard of care and that the smaller RT fields used in more recent studies are likely associated with a lower risk for the development of potentially lethal RT-related late effects.

In patients with a positive interim PET after 2 cycles of ABVD, improved tumor control in comparison with continued ABVD therapy could be documented after treatment intensification with 2 cycles of escalated BEACOPP before limited-field RT.^6^ Therefore, treatment intensification with escalated BEACOPP should be considered when metabolic remission has not been achieved after 2 cycles of ABVD.

In addition to the optimal use of interim PET, treatment optimization by using targeted drugs such as the CD30-directed antibody–drug conjugate BV or the anti-PD-1 antibody nivolumab is currently being evaluated in patients with intermediate-stage cHL. Early results of a study investigating the combination of AVD (doxorubicin, vinblastine, dacarbazine) chemotherapy and BV followed by involved-site RT indicated excellent response rates and a promising short-term tumor control.^27^ An ongoing study by the GHSG evaluates the combination of AVD chemotherapy and nivolumab (NCT03004833). Data from this study are not yet available.

Major controversy:

4 cycles of ABVD + limited-field RT (25) vs ABVD only in patients with a negative interim PET (6)

### Advanced stages

Advanced-stage cHL (Table 1) is most commonly treated either with escalated BEACOPP or ABVD chemotherapy. Although the available data demonstrate a consistently better tumor control with escalated BEACOPP, this more aggressive regimen does not represent the standard of care for all institutions and study groups.^28^ This is due to the different weighting of the higher acute toxicity, the increased risk of treatment-related late effects, and controversy regarding the effect on OS.^29–32^ The focus of current trials is to reduce toxicity without compromising efficacy.

The randomized GHSG HD15 and HD18 trials demonstrated that the number of cycles of escalated BEACOPP could be reduced from 6 to 4 in patients with a negative interim PET after 2 cycles and that consolidation RT is only necessary in case of PET-positive residual lymphoma >2.5 cm at the end of chemotherapy.^7,33^ The HD18 trial indicated an excellent 5-year OS rate of 95% and a significant reduction of severe acute hematological and nonhematological toxicities. The ongoing randomized GHSG HD21 study investigates whether a further reduction of toxicity can be realized with a BV-containing BEACOPP variant termed BrECADD (BV, etoposide, cyclophosphamide, doxorubicin, dacarbazine, dexamethasone). This protocol was shown to induce promising response rates and a decreased rate of severe acute toxicities in a previous phase II study.^9^ The randomized LYSA AHL2011 trial evaluated whether patients with a negative PET after 2 cycles of escalated BEACOPP could complete treatment with 4 cycles of ABVD without loss of tumor control. Patients assigned to the standard arm received a total of 6 cycles of escalated BEACOPP. After a median observation time of 50 months, 5-year PFS rates did not differ. Thus, it appears possible to switch from escalated BEACOPP to ABVD on the basis of a negative interim PET after 2 cycles of escalated BEACOPP.^34^

Groups using ABVD as initial therapy have also aimed at reducing toxicity, especially in patients with a negative interim PET. In the international RATHL trial for advanced-stage cHL, all patients initially received 2 cycles of ABVD. In the experimental arm, bleomycin was not given in the remaining 4 cycles in patients with a negative interim PET. The results showed 3-year PFS rates of 85.7% and 84.4%, respectively, in the standard ABVD and AVD groups. The rate of adverse events was reduced in patients treated with AVD in cycles 3 through 6. This was not only true for pulmonary toxicities but the proportion of patients who developed fatigue or episodes of febrile neutropenia was also lower.^8^ Thus, bleomycin is redundant in advanced-stage patients who have achieved a complete metabolic response after 2 cycles of chemotherapy. In patients with advanced cHL who have a negative PET at the end of ABVD chemotherapy, the Gruppo Italiano Terapie Innovative Linfomi (GITIL)/FIL HD 0607 trial demonstrated that consolidation RT to initially bulky lesions can be omitted without a decrease in tumor control at 3 years.^35^ Attempts have also been made to improve treatment outcomes by modifications of the ABVD chemotherapy backbone and intensification of therapy in patients without complete metabolic response according to an interim PET. The international ECHELON-1 study randomly compared standard ABVD and the combination of AVD and BV (A-AVD). The trial revealed a slightly improved modified 2-year PFS rate for the novel combination comprising BV. However, the 4.9% improvement in tumor control came at the cost of more toxicity including polyneuropathy, and neutropenia necessitating G-CSF. More follow-up is needed for more sound conclusions on the role of A-AVD in advanced cHL but comparability with other studies is impeded by the use of a modified PFS as primary endpoint and the lack of data on conventional PFS.^10^ Other nonrandomized trials investigated interim PET-guided treatment intensification. Patients with a positive PET after 2 cycles of ABVD continued treatment with escalated BEACOPP and IGEV (ifosphamide, gemcitabine, vinorelbine) followed by high-dose chemotherapy and ASCT, respectively.^8,35–37^ These approaches appeared to result in better outcomes than continued ABVD therapy compared to historical controls, but no randomized comparisons have been conducted to date.

For both escalated BEACOPP and ABVD, the major goal is the optimization of the long-term risk-benefit ratio. A more effective risk allocation would be very helpful for this and may be achieved by the inclusion of novel biomarkers such as biological assays or the evaluation of baseline PET parameters in future risk prediction models.^38^ The potential benefit of more intensive initial therapy appears greatest for patients with high-risk features at presentation, and more accurate methods for identifying these could allow appropriate stratification of the initial chemotherapy.

**Table 2:**
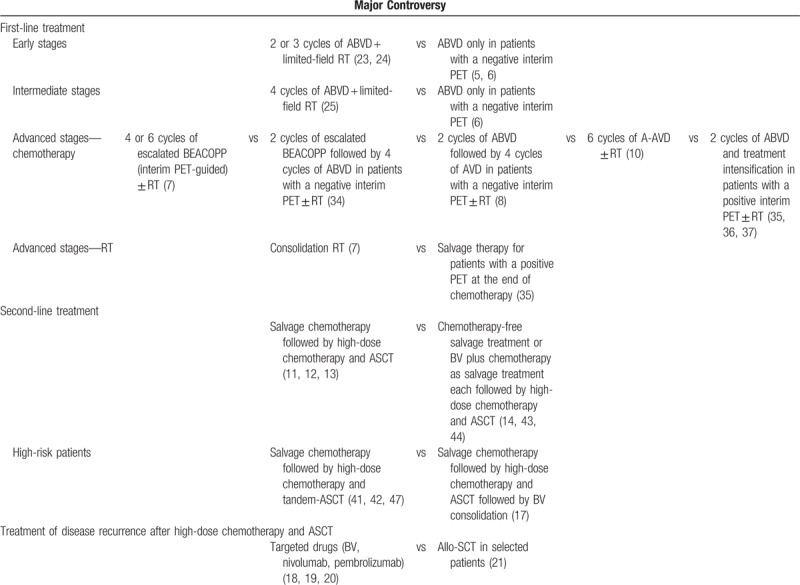
Major Controversies in the Treatment of Hodgkin Lymphoma

The role of RT after completion of chemotherapy in advanced-stage patients remains uncertain. Consolidation RT is normally applied if larger residual lymphoma with metabolic activity is detected after the end of chemotherapy, but no randomized study has demonstrated the benefit of such an approach.^7,33^ Some studies did not pre-specify RT after completion of chemotherapy and rather proposed salvage approaches in patients with a positive PET at the end of chemotherapy.^35^ Conversely, consolidation RT does not improve the outcome of patients with negative PET after completion of chemotherapy.^35^

Major controversies:

Chemotherapy: 4 or 6 cycles of escalated BEACOPP (interim PET-guided) ± RT (7) vs 2 cycles of escalated BEACOPP followed by 4 cycles of ABVD in patients with a negative interim PET ± RT (34) vs 2 cycles of ABVD followed by 4 cycles of AVD in patients with a negative interim PET ± RT (8) vs 6 cycles of A-AVD ± RT (10) vs 2 cycles of ABVD and treatment intensification in patients with a positive interim PET ± RT (35, 36, 37)

RT: consolidation RT (7) vs salvage therapy for patients with a positive PET at the end of chemotherapy (35)

## Second-line treatment

The standard of care for patients with relapsed cHL consists of salvage chemotherapy followed by high-dose chemotherapy and ASCT. Different salvage protocols including DHAP (dexamethasone, high-dose ara-c, cisplatin), ICE (ifosphamide, carboplatin, etoposide), BeGEV (bendamustine, gemcitabine, vinorelbine) and IGEV have shown antitumor activity and the capacity to mobilize stem cells.^15,39,40^ The BEAM protocol (BCNU, etoposide, ara-c, melphalan) is the most commonly used conditioning protocol before ASCT.

Recent studies for patients with disease recurrence after first-line treatment have investigated whether tandem-ASCT or the incorporation of novel drugs may optimize the outcome, especially for high-risk patients. A strategy including tandem-ASCT was proposed by the GELA. This approach gave a freedom from second failure rate of 46% at 5 years and 41% at 10 years in patients with high-risk relapse or refractory disease (primary disease progression, relapse <12 months after the end of first-line treatment, stage III or IV at relapse, relapse within previously irradiated sites).^41,42^ The randomized AETHERA study evaluated the role of BV consolidation after high-dose chemotherapy and ASCT in patients presenting with poor-risk features (primary disease progression, relapse <12 months after the end of first-line treatment, extranodal disease at relapse). After a median follow-up of 30 months, the median PFS with BV was significantly better than with placebo (42.9 months vs 24.1 months). Overall survival was the same in both groups most likely due to the use of BV as salvage therapy in patients from the placebo arm relapsing during observation.^17^ Based on the results of this study, BV was approved for consolidation treatment after high-dose chemotherapy and ASCT in patients with poor-risk relapsed cHL.

Brentuximab vedotin was also investigated as salvage treatment before high-dose chemotherapy and ASCT, given as single agent or in combination with either chemotherapy or the anti-PD-1 antibody nivolumab. A phase II study included a total of 45 patients with relapsed cHL who received 2 cycles of single-agent BV. Each cycle consisted of 4 weekly infusions at 1.2 mg/kg. A negative PET after 8 weeks was documented for 27% of patients. These patients proceeded to high-dose chemotherapy and ASCT and had a 2-year event-free survival rate of 92%.^14^ The combination of bendamustine and BV was evaluated in a phase I/II study which enrolled 55 patients including 28 with primary refractory disease and 27 with relapsed disease. Thirty-nine of the 53 patients (74%) evaluable for response achieved a complete remission (CR) and 40 patients underwent ASCT. Long-term results of this trial are pending.^43^ The combination of BV and nivolumab was evaluated in a phase I/II study comprising 62 patients with relapsed cHL. Up to 4 21-day cycles were administered. An interim analysis demonstrated a CR according to the Lugano classification in 62% of study participants. Overall, 42 patients proceeded to high-dose chemotherapy and ASCT immediately after salvage therapy with the combination of BV and nivolumab. No new safety concerns were reported.^44^ Follow-up analyses will allow conclusions regarding long-term tumor control after salvage therapy with BV and nivolumab before high-dose chemotherapy and ASCT.

In addition to the optimization of salvage treatment before high-dose chemotherapy and ASCT, a number of further questions regarding the therapy of patients with relapsed cHL are still unanswered. For instance, the most appropriate procedure in patients who still have a positive PET after salvage therapy is undefined. Treatment with high-dose chemotherapy and ASCT can be completed as intended, but switching to another noncross-resistant salvage regimen or the application of localized RT to achieve a complete metabolic response should also be discussed, as PET-negativity before high-dose chemotherapy and ASCT was shown to be a predictor for better long-term tumor control.^45,46^ Tandem-ASCT may also represent an option in patients with a positive PET after salvage therapy as this approach appears to be associated with an improved tumor control in comparison with a single ASCT.^47^ The identification of low-risk patients with cHL recurrence who are possibly treated adequately without high-dose chemotherapy and ASCT is another unsolved issue.

Major controversies:

Standard-risk patients: salvage chemotherapy followed by high-dose chemotherapy and ASCT (11, 12, 13) vs chemotherapy-free salvage treatment or BV plus chemotherapy as salvage treatment each followed by high-dose chemotherapy and ASCT (14, 43, 44)

High-risk patients: salvage chemotherapy followed by high-dose chemotherapy and tandem-ASCT (41, 42, 47) vs salvage chemotherapy followed by high-dose chemotherapy and ASCT followed by BV consolidation (17)

## Treatment of disease recurrence after high-dose chemotherapy and ASCT

Long-term cure is uncommon in cHL patients who relapse after high-dose chemotherapy and ASCT and for those who are unable to undergo this procedure, either due to advanced age, comorbidities, or lack of adequate tumor response. Fortunately, several new active treatment options for this situation have become available. Brentuximab vedotin is approved for the treatment of patients with disease recurrence after high-dose chemotherapy and ASCT. The approval has been granted on the basis of results from a pivotal phase II study including 102 patients. The antibody–drug conjugate was given for a maximum of 16 21-day cycles. The overall response rate (ORR) was 75%.^18^ At 5 years, the OS rate was 41%. However, only few patients achieved continuous remission without subsequent treatment after BV.^48^ The most effective treatment approach for patients who relapse after BV consists of the anti-PD-1 antibodies nivolumab and pembrolizumab. Both drugs were shown to be highly active in heavily pretreated cHL patients. The ORR with nivolumab was 69% and the median PFS 14.7 months.^49^ Similar data were obtained with pembrolizumab.^20^ Both drugs were therefore approved for the treatment of cHL patients with disease recurrence after high-dose chemotherapy followed by ASCT and BV. Whether the administration of an anti-PD-1 antibody before BV may be better than sequential use according to the current approval should be subject to future studies.

Allogeneic stem cell transplantation (allo-SCT) represents a potentially curative treatment modality for cHL patients with recurrent disease after high-dose chemotherapy and ASCT. A phase II study evaluated reduced-intensity conditioning with fludarabine and melphalan. The 4-year PFS rate after allo-SCT was 24%.^21^ Given this rather disappointing tumor control and the risk of transplantation-associated adverse events such as severe graft-versus-host disease on the one hand and the excellent efficacy of novel drugs on the other hand, the possible future role for allo-SCT in the treatment algorithm of relapsed cHL remains uncertain.

Major controversy:

Targeted drugs (BV, nivolumab, pembrolizumab) (18, 19, 20) vs allo-SCT in selected patients (21)

## Summary

Although the majority of patients can be cured with risk-adapted first-line treatment, further optimization of cHL treatment is necessary. The major goal in newly diagnosed cHL includes the reduction of treatment-related toxicity. This might be achieved by the use of PET-guided approaches and the implementation of targeted drugs. The use of targeted drugs is also being evaluated in patients with disease recurrence after first-line treatment and in those relapsing after high-dose chemotherapy and ASCT.
